# Synthesis under Microwave Irradiation of [1,2,4]Triazolo[3,4-*b*][1,3,4]thiadiazoles and Other Diazoles Bearing Indole Moieties and Their Antimicrobial Evaluation

**DOI:** 10.3390/molecules16108244

**Published:** 2011-09-28

**Authors:** Sobhi M. Gomha, Sayed M. Riyadh

**Affiliations:** Department of Chemistry, Faculty of Science, University of Cairo, Giza 12613, Egypt; Email: riyadh1993@hotmail.com (S.M.R.)

**Keywords:** [1,2,4]triazolo[3,4-*b*][1,3,4]thiadiazoles, diazoles, Schiff’s bases, microwave irradiation and antimicrobial activity

## Abstract

Microwave-assisted synthesis of some novel compounds, namely, 3-(2-methyl-1*H*-indol-3-yl)-6-aryl-[1,2,4]triazolo[3,4-*b*][1,3,4]thiadiazoles **5a,b** was accomplished via bromination of 2-methyl-3-[4-(arylideneamino)-5-mercapto-4*H*-[1,2,4]triazol-3-yl]-1*H*-indoles **3a,b**. Also, new [1,3,4]thiadiazoles **12a,b,** [1,2,4]triazoles **15a,b** and [1,3,4]oxadiazoles **19a,b**, with indole moieties, were prepared by cyclization of 1-[(2-methyl-1*H*-indole)-3-carbonyl]thiosemicarbazides **8a,b** under microwave irradiation using different reaction conditions. Moreover, reaction of acid hydrazide **7** with ethyl 2-(*N*-phenylhydrazono)-3-oxobutanoate (**20**) gave the respective phenylhydrazonopyrazole derivative **21** under the reaction conditions employed. The structures of the synthesized compounds were assigned based on elemental analyses and spectral data (IR, ^1^H-NMR, ^13^C-NMR, MS). The antifungal and antibacterial activities of the new products were also evaluated.

## 1. Introduction

Reports of the synthesis of [1,2,4]triazolo[3,4-*b*][1,3,4]thiadiazole derivatives have been recently published [1,2,3,4,5]. A route for the construction of such ring systems involves reactions of 4-amino-5-mercapto-4*H*-[1,2,4]triazoles with aromatic carboxylic acids in the presence of suitable dehydrating agents [1,2,3,4,5]. Another route involves oxidative cyclization of hydrazones [6]. In view of these findings and in continuation of our interest in the utility of 4-amino-5-mercapto-4*H*-[1,2,4]triazoles as precursors for the synthesis of heterocycles [7,8], we wish to report herein a simple and convenient route for the synthesis of [1,2,4]triazolo[3,4-*b*][1,3,4]thiadiazole derivatives. This route involves bromination of hitherto unreported 4-(arylideneamino)-5-mercapto-4*H*-[1,2,4]triazoles with concurrent elimination of hydrogen bromide. The interest in developing syntheses of [1,2,4]triazolo[3,4-*b*] [1,3,4]thiadiazoles is due to the fact that these compounds are potent antioxidants and anticancer agents [9] and have other pharmacological activities [10]. On the other hand, the chemistry of indole derivatives has been the subject of much interest in recent years due to the use of this ring system as the core structure in many heterocyclic compounds covering wide range of pharmacological applications [11,12]. Merging of these two bioactive components, [1,2,4]triazolo[3,4-*b*][1,3,4]-thiadiazole and indole, in one system affords a compact structure with promising potential antimicrobial activities. 

## 2. Results and Discussion

**Scheme 1:**
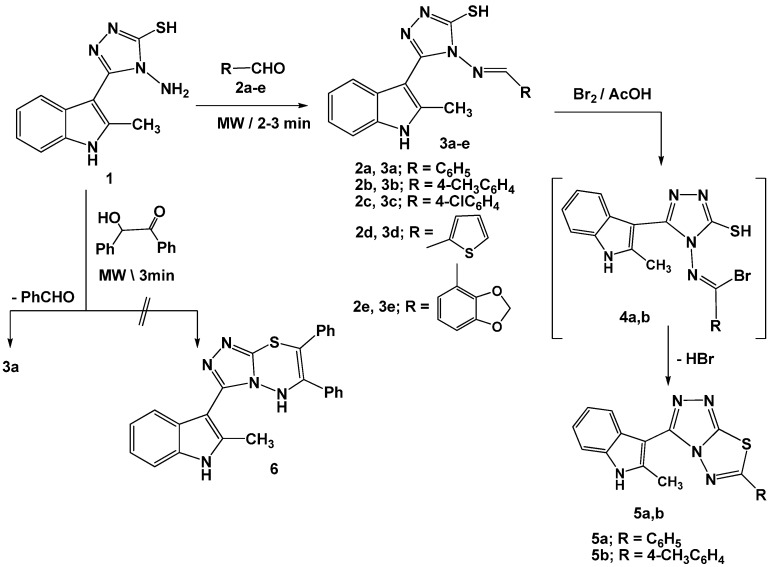
Synthesis of [1,3,4]triazolo[3,4-*b*][1,3,4]thiadiazoles.

2-Methyl-3-[4-amino-5-mercapto-4*H*-[1,2,4]triazol-3-yl]-1*H*-indole (**1**) [7] was condensed under microwave irradiation with different aldehydes such as substituted benzaldehydes **2a**-**c**, 2-thiophenaldehyde (**2d**), and benzo[*d*]-[1,3]dioxole-4-carbaldehyde (**2e**) in dimethylformamide, in the presence of catalytic amount of HCl, to give the corresponding 4-arylideneamino-[1,2,4]triazole Schiff’s base derivatives **3a**-**e**, respectively (Scheme 1). On the other hand, reaction of **1** with benzoin in ethanol in the presence of KOH gave the corresponding benzylidene derivative **3a** instead of the expected [1,2,4]triazolo[3,4-*b*][1,3,4]thiadiazine derivative **6** (Scheme 1).

The structures of the isolated products **3a**-**e** were substantiated based on elemental analyses and spectral data. The ^1^H-NMR spectra revealed, in each case, a singlet signal at *δ* 13.76-13.81 ppm (exchangeable with D_2_O) assignable to a thiol proton [13,14,15] and another singlet signal at *δ* 9.91-10.0 ppm which was attributed to the azomethine proton [13,14,15]. Room temperature (25 °C) bromination of 4-arylideneamino[1,2,4]triazole derivatives **3a**,**b** in acetic acid in the presence of anhydrous sodium acetate afforded the respective [1,2,4]triazolo[3,4-*b*][1,3,4]thiadiazoles **5a**,**b** via dehydrobromination from the non-isolable intermediates **4a,b** (cf. Scheme 1).

**Scheme 2:**
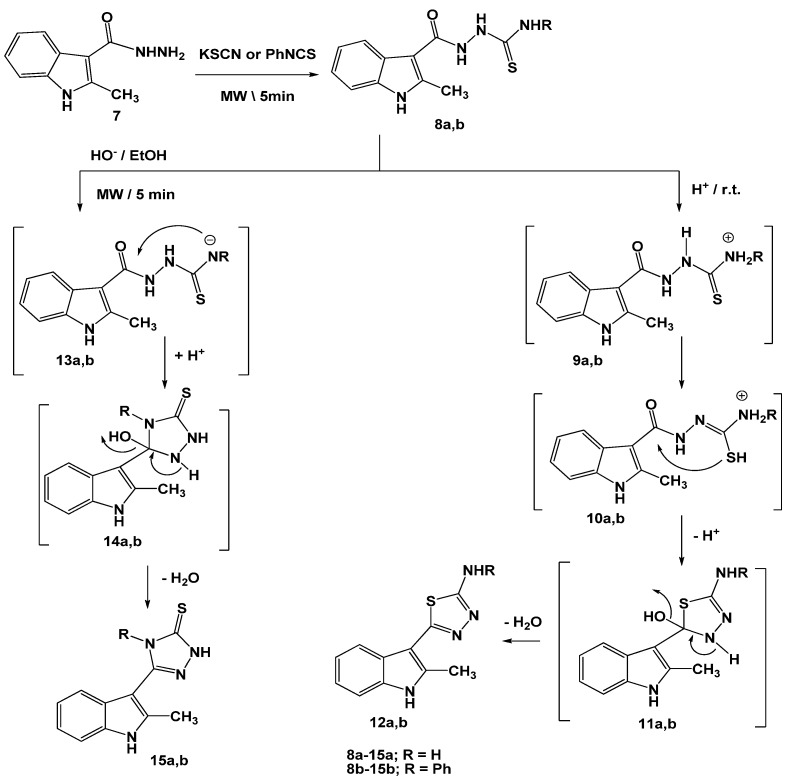
Reactions of thiosemicarbazides **8a**,**b** under different pH conditions.

The synthetic utility of 1-[(2-methyl-1*H*-indole)-3-carbonyl]thiosemicarbazides **8a,b** prepared from acid hydrazide **7** [13,16] and potassium thiocyanate or phenyl isothiocyanate was investigated. Thus, treatment of **8a,b** with concentrated sulfuric acid at room temperature (25 °C) gave the respective[1,3,4]thiadiazole derivatives **12a,b**. The reaction proceeded via intramolecular nucleophilic attack of thiol group into carbonyl group [17] to give intermediates **11a**,**b**. Dehydration of these intermediates **11a,b** under the employed reaction conditions gave the isolated products **12a,b** (Scheme 2). Protonation of N-4 (NHR) in the thiosemicarbazide moiety under acidic condition (intermediates **9a,b**) reduces its nucleophilicity and increases the nucleophilicity of the thiol group [17].

**Scheme 3:**
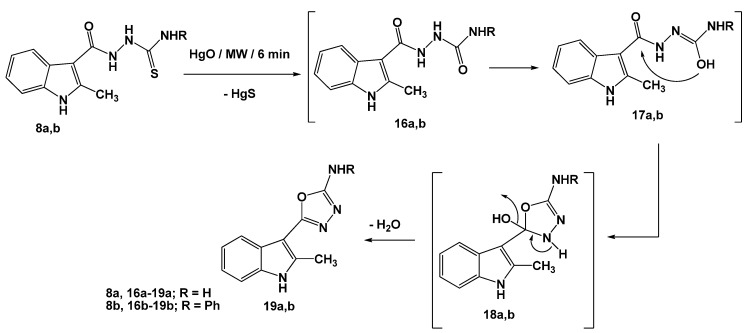
Reactions of thiosemicarbazides **8a**,**b** with HgO.

Changes of the pH of the reaction mixture led to the formation of different ring systems. Thus, reactions of **8a,b** with potassium hydroxide, as basic catalyst, led to the formation of [1,2,4]triazoles **15a,b**. The reaction started by abstraction of a hydrogen atom from the NH-R group to increase its nucleophilicity [17] (intermediates **13a,b**) followed by its attack on the carbonyl group with concurrent dehydration of the non-isolable intermediates **14a,b** (cf. Scheme 2) to give isolable products **15a,b**. The structures of the isolated products **12a,b**, and **15a,b** were established on the basis of elemental analyses and spectral data (see Experimental). On the other hand, irradiation of **8a,b** with mercuric oxide furnished the corresponding [1,3,4]oxadiazoles **19a,b**, via elimination of mercuric sulfide [17] from the non-isolable intermediates **16a,b**. Nucleophilic attack of hydroxyl group on the carbonyl carbon gave intermediates **18a,b** followed by loss of a water molecule to afford the isolated products **19a,b** (Scheme 3).

**Scheme 4:**
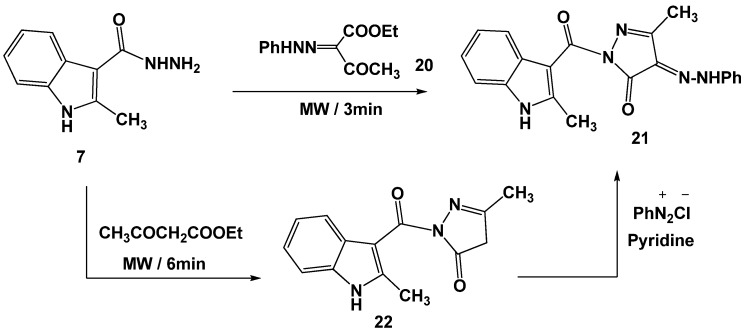
Synthesis of phenylhydrazonopyrazolinone derivative **21**.

Next, we studied the reactivity of hydrazone derivatives which are widely used as intermediates for the synthesis of a large number of heterocyclic compounds [18]. Thus, treatment of (2-methyl-1*H*-indol-3-yl)-3-acid hydrazide (**7**) with ethyl 2-(*N*-phenylhydrazono)-3-oxobutanoate (**20**) [19] in DMF under microwave irradiation in the presence of a catalytic amount of HCl afforded the corresponding 3-[(3-methyl-4-phenylhydrazono-5-oxopyrazolin-1-yl)carbonyl]-2-methyl-1*H*-indole (**21**) via elimination of ethanol and water molecules (Scheme 4).

The structure of the latter product was confirmed by the appearance of the hydrazone NH band at 3358 cm^−1^ in its IR spectrum [20]. The ^1^H- NMR spectrum also revealed two singlet signals at *δ* 11.20 and 13.11 ppm (D_2_O exchangeable) assignable to the NH of the pyrrole ring and hydrazone group [20], respectively. An alternative synthesis of **21** was carried out as outlined in Scheme 4. Thus, reaction of acid hydrazide **7** with ethyl 3-oxobutanoate under microwave irradiation for 6 min. gave pyrazolinone derivative **22**. Coupling of **22** with diazotized aniline in pyridine afforded the corresponding hydrazone derivative **21**, typical in all respects (mp., mixed mp., and IR) to that obtained from acid hydrazide **7** and **20** (cf. Scheme 4). 

## 3. Antimicrobial Activity

The *in vitro* antimicrobial activities of the synthesized products **3a-e**, **5a,b**, **8b**, **12a,b**, **15a**,**b**, **19a,b**, and **21** were screened against the Gram-positive bacteria *Staphylococcus aureus* (*S. aureus*), *Bacillus subtilis* (*B. subtilis*), Gram-negative bacteria *Escherichia coli* (*E. coli*) and the fungus *Candida albicans* (*C. albicans*) under the same conditions using trimethoprim as reference. The bacteria and fungus were subjected to susceptibility testing on Muller-Hinton agar medium by the disc agar diffusion method [21,22]. The results are summarized in Table 1.

**Table 1:** Antimicrobial activity of the tested compounds.

Sample number	Inhibition zone diameter (mm/mg sample)			
	Gram-positive Bacteria		Gram-negative Bacteria	Fungus
	*S. aureus*	*B. subtilis*	*E. coli*	*C. albicans*
**3a**	15	14	16	17
**3b**	12	11	-	-
**3c**	14	13	16	15
**3d**	-	16	-	-
**3e**	12	16	18	15
**5a**	17	15	18	14
**5b**	16	15	17	18
**8b**	12	15	-	-
**12a**	13	12	-	-
**12b**	-	-	12	10
**15a**	12	-	13	-
**15b**	-	14	-	-
**19a**	-	12	11	10
**19b**	15	-	9	11
**21**	13	15	17	16
**Trimethoprim**	19	19	21	21
(-) No inhibition zone.				

The following conclusions may be drawn from the results:

**a)** Compounds **3a**, **5a**, **5b**, and **19b** exhibited high inhibitory effect against *S. aureus*, while compounds **3b**, **3c**, **3e**, **8b**, **12a**, **15a**, and **21** have moderate inhibitory effect against same bacterium. On the other hand, compounds **3d**, **12b**, **15b**, and **19a** have no inhibitory effect against *S. aureus*.
**b)** Compounds **3a**, **3d**, **3e**, **5a**, **5b**, **8b**, and **21** revealed high inhibitory activity against *B. subtilis*, while compounds **3b**, **3c**, **12a**, **15b**, and **19a** have moderate inhibitory activity against this species and compounds **12b**, **15a**, and **19b** have no activity against *B. subtilis*.
**c)** Compounds **3a**, **3c**, **3e, 5a**, **5b**, and **21** were effective against *E. coli* and *C. albicans*. Other compounds, **12b**, **15a**, **19a**, and **19b** have moderate inhibitory activity against *E. coli* and *C. albicans*. Compounds **3b**, **3d**, **8b**, **12a**, and **15b** are inactive against *E. coli* and *C. albicans*.

## 5. Experimental

Melting points were measured on an Electrothermal IA 9000 series digital melting point apparatus. The IR spectra were recorded in potassium bromide discs on a Pye Unicam SP 3300 and Shimadzu FT IR 8101 PC infrared spectrophotometers. The NMR spectra were recorded on a Varian Mercury VX-300 NMR spectrometer operating at 300 MHz (^1^H-NMR) or 75 MHz (^13^C-NMR) and run in deuterated dimethylsulphoxide (DMSO-*d*_6_). Chemical shifts were related to that of the solvent. Mass spectra were recorded on a Shimadzu GCMS-QP1000 EX mass spectrometer at 70 eV. Elemental analyses (using a German made Elementar vario LIII CHNS analyzer) and the biological evaluation of the products were carried out at the Microanalytical Centre of Cairo University, Giza, Egypt. All reactions were followed by TLC (silica gel, aluminum Sheets 60 F254, Merck). Irradiation was done in a domestic microwave oven (R-658WM, 2500 MHz, 400 W). The reactions were carried out in a closed Teflon vessel which was placed at the center of the oven for irradiation. 2-Methyl-3-[4-amino-5-mercapto-4*H*-[1,2,4]triazol-3-yl]-1*H*-indole (**1**) [7], 2-methyl-1*H*-indole-3-carbohydrazide (**7**) [16], 1-[(2-methyl-1*H*-indole)-3-carbonyl]thiosemicarbazide (**8a**) [16] and ethyl 2-(*N*-phenylhydrazono)-3-oxobutanoate (**20**) [19] were prepared as previously reported in the respective literature.

### 5.1. Reaction of 2-methyl-3-[4-amino-5-mercapto-4H-[1,2,4]triazol-3-yl]-1H-indole *(**1**)* with aldehydes

A mixture of **1** (0.245 g, 1 mmol), and the appropriate aldehyde (1 mmol) in DMF (5 mL) containing a few drops of concentrated HCl was irradiated by MW at 400 Watt in a closed Teflon vessel until all the starting material was consumed (2–3 min., as monitored by TLC). Excess of solvent was removed under reduced pressure and the reaction mixture was added to crushed ice. The product separated was filtered, washed with water, dried and recrystallized from the proper solvent to give compounds **3a-e**.

*2-Methyl-3-[4-(phenylmethyleneamino)-5-mercapto-4H-[1,2,4]triazol-3-yl]-1H-indole* (**3a**): Yield 84%; pale yellow microcrystals (from ethanol); mp = 352 °C. IR: *v* 1627 (CH=N), 3398 (NH) cm^−1^. ^1^H-NMR: *δ* 2.51 (s, 3H, CH_3_), 6.98-7.92 (m, 9H, ArH), 9.98 (s, 1H, CH=N), 11.16 (s, 1H, D_2_O exchangeable, NH), 13.81 (s,1H, D_2_O exchangeable, SH); ^13^C-NMR: *δ* 14.75 (CH_3_), 111.15, 117.75, 120.29, 120.85, 121.86, 123.75, 124.33, 125.75, 127.16, 127.31, 129.73, 132.61, 148.13, 156.47, 160.11 (Ar-C); MS *m/z* (%): 334 (M^+^ +1, 10), 333 (M^+^, 10), 229 (100), 155 (32), 103 (35), 77 (23). Anal. Calcd for C_18_H_15_N_5_S (333.10): C, 64.84; H, 4.53; N, 21.01; S, 9.62. Found C, 64.54; H, 4.35; N, 20.84; S, 9.48%.

*2-Methyl-3-[4-(4-methylphenylmethyleneamino)-5-mercapto-4H-[1,2,4]triazol-3-yl]-1H-indole* (**3b**): Yield 85%; pale yellow microcrystals (from ethanol); mp = 338 °C. IR: *v* 1627 (CH=N), 3398 (NH) cm^−1^. ^1^H-NMR: *δ* 2.37 (s, 3H, CH_3_), 2.51 (s, 3H, CH_3_), 6.85–7.83 (m, 8H, ArH). 9.96 (s, 1H, CH=N), 11.19 (s, 1H, D_2_O exchangeable, NH), 13.78 (s,1H, D_2_O exchangeable, SH). MS *m/z* (%): 348 (M^+^ +1, 12), 347 (M^+^, 36), 229 (100), 197 (32), 155 (31), 117 (79), 102 (8), 77 (16). Anal. Calcd for C_19_H_17_N_5_S (347.12): C, 65.68; H, 4.93; N, 20.16; S, 9.23%. Found C, 65.56; H, 4.85; N, 19.69; S, 9.11%.

*2-Methyl-3-[4-(4-chlorophenylmethyleneamino)-5-mercapto-4H-[1,2,4]triazol-3-yl]-1H-indole* (**3c**): Yield 82%; pale yellow microcrystals (from ethanol/dioxane); mp = 358 °C. IR: *v* 1628 (CH=N), 3401 (NH) cm^−1^. ^1^H-NMR: *δ* 2.51 (s, 3H, CH_3_), 6.87-7.93 (m, 8H, ArH), 9.99 (s, 1H, CH=N), 11.51 (s, 1H, D_2_O exchangeable, NH), 13.76 (s, 1H, D_2_O exchangeable, SH). MS *m/z* (%): 368 (M^+^ +1, 11), 367 (M^+^, 36), 229 (100), 155 (38), 117 (59), 77 (41). Anal. Calcd for C_18_H_14_ClN_5_S (367.07): C, 58.77; H, 3.84; N, 19.04; S, 8.72%. Found C, 58.65; H, 3.82; N, 18.78; S, 8.88%.

*2-Methyl-3-[4-(thiophen-2-ylmethyleneamino)-5-mercapto-4H-[1,2,4]triazol-3-yl]-1H-indole* (**3d**): Yield 84%; pale yellow microcrystals (from ethanol); mp = 325 °C. IR: *v* 1624 (CH=N), 3409 (NH) cm^−1^. ^1^H-NMR: *δ* 2.51 (s, 3H, CH_3_), 6.47-7.60 (m, 7H, ArH), 10.00 (s, 1H, CH=N), 10.72 (s, 1H, D_2_O exchangeable, NH), 13.80 (s, 1H, D_2_O exchangeable, SH); ^13^C-NMR: *δ* 14.38 (CH_3_), 112.05, 118.45, 120.29, 121.05, 122.36, 123.75, 124.73, 125.75, 128.16, 129.31, 132.73, 137.61, 150.13, 156.77, 160.16 (Ar-C); MS *m/z* (%): 339 (M^+^, 38), 229 (100), 197 (12), 156 (12), 128 (10), 109 (8), 77 (7). Anal. Calcd for C_16_H_13_N_5_S_2_ (339.06): C, 56.61; H, 3.86; N, 20.63; S, 18.89%. Found C, 56.51; H, 3.80; N, 20.44; S, 18.76%.

*2-Methyl-3-[4-(benzo[d][1,3]dioxol-4-ylmethyleneamino)-5-mercapto-4H-[1,2,4]triazol-3-yl]-1H-indole* (**3e**): Yield 84%; yellow microcrystals (from DMF); mp = 330 °C. IR: *v* 1628 (CH=N), 3402 (NH) cm^−1^; ^1^H-NMR: *δ* 2.50 (s, 3H, CH_3_), 5.96 (s, 2H, CH_2_), 6.14-7.76 (m, 7H, ArH), 9.61 (s, 1H, CH=N), 11.17 (s, 1H, D_2_O exchangeable, NH), 13.76 (s,1H, D_2_O exchangeable, SH). MS *m/z* (%): 378 (M^+^ +1, 10), 377 (M^+^, 43), 229 (100), 197 (9), 155 (12), 146 (82), 77 (10). Anal. Calcd for C_19_H_15_N_5_O_2_S (377.09): C, 60.46; H, 4.01; N, 18.56; S, 8.50%. Found C, 60.51; H, 3.86; N, 18.40; S, 8.62%.

### 5.2. Reaction of 2-methyl-3-(4-amino-5-mercapto-4H-[1,2,4]triazol-3-yl)-1H-indole *(**1**)* with 2-hydroxy-1,2-diphenylethanone (benzoin)

To a solution of **1** (0.245g, 1 mmol) in DMF (5 mL) containing a solution of potassium hydroxide (0.084 g, 1.5 mmol) in water (1 mL), benzoin (0.212 g, 1 mmol) was added in a closed Teflon vessel. The reaction mixture was irradiated by MW at 400 Watt for 3 min. then poured into crushed ice. The reaction product was acidified with dilute hydrochloric acid. The solid product was collected by filtration, washed with water and finally recrystallized from ethanol to give a product identical in all respects (mp, mixed mp and IR spectra) with product **3a** which was obtained from reaction of **1** with benzaldehyde.

### 5.3. Synthesis of 3-(2-methyl-1H-indol-3-yl)-6-aryl-[1,2,4]triazolo[3,4-b][1,3,4]thiadiazoles ***5a,b***

Bromine (0.44 g, 5.5 mmol) in acetic acid (5 mL) was added dropwise to a stirred mixture of the appropriate Schiff’s bases **3a,b** (5 mmol) and sodium acetate (1.2 g, 15 mmol) in acetic acid (30 mL). The reaction mixture was stirred for 12 h at room temperature. The mixture was then poured onto ice-cold water (250 mL). The solid that precipitated was filtered off, washed with 5% sodium bicarbonate solution and then with water, dried and crystallized from ethanol to give the respective [1,2,4]triazolo[3,4-*b*][1,3,4]thiadiazoles **5a,b**.

*3-(2-Methyl-1H-indol-3-yl)-6-phenyl-[1,2,4]triazolo[3,4-b][1,3,4]thiadiazole* (**5a**): Yield 74%; yellow crystals; mp = 228 °C. IR: *v* 3407 (NH) cm^−1^. ^1^H-NMR: *δ* 2.54 (s, 3H, CH_3_), 6.87–7.89 (m, 9H, ArH), 11.21 (s, 1H, D_2_O exchangeable, NH); ^13^C-NMR: *δ* 14.33 (CH_3_), 112.15, 118.75, 120.26, 121.05, 122.36, 123.75, 124.33, 125.75, 127.16, 127.31, 129.73, 130.61, 153.13, 156.57, 159.84 (Ar-C); MS *m/z* (%): 332 (M^+^ +1, 9), 331 (M^+^, 42), 243 (53), 158 (100), 102 (26), 77 (34). Anal. Calcd for C_18_H_13_N_5_S (331.09): C, 65.24; H, 3.95; N, 21.13; S, 9.68%. Found C, 65.14; H, 3.91; N, 21.11; S, 9.54%.

*3-(2-Methyl-1H-indol-3-yl)-6-(4-methylphenyl)-[1,2,4]triazolo[3,4-b][1,3,4]thiadiazole* (**5b**). Yield 78%; yellow crystals; mp = 212 °C. IR: *v* 3409 (NH) cm^−1^. ^1^H-NMR: *δ* 2.32 (s, 3H, Ar-CH_3_), 2.58 (s, 3H, CH_3_), 6.86–7.88 (m, 8H, Ar-H), 11.20 (s, 1H, D_2_O exchangeable, NH); ^13^C-NMR: *δ* 14.35 (CH_3_), 35.65 (Ar-CH_3_), 111.77, 117.75, 120.16, 120.95, 121.36, 122.75, 123.93, 124.85, 127.16, 128.71, 129.73, 130.61, 152.16, 155.53, 159.14 (Ar-C); MS *m/z* (%): 346 (M^+^ +1, 7), 345 (M^+^, 33), 243 (43), 158 (100), 77 (29). Anal. Calcd for C_19_H_15_N_5_S (345.10): C, 66.07; H, 4.38; N, 20.27; S, 9.28%. Found C, 65.97; H, 4.18; N, 20.20; S, 9.33%.

### 5.4. Synthesis of 1-[(2-methyl-1H-indole)-3-carbonyl]phenylthiosemicarbazide *(**8b**)*

A mixture of acid hydrazide **7** (1.89, 10 mmol) and phenyl isothiocyanate (1.35 g, 10 mmol) in DMF (5 mL) was irradiated by MW at 400 Watt for 5 min. in a closed Teflon vessel (monitored by TLC). After cooling, chloroform (10 mL) was added and the reaction mixture was stirred for 5 min., then filtered and the crude product was crystallized from ethanol to yield phenylthiosemicarbazide derivative **8b**. Yield 87%; pale yellow microcrystals; mp = 165 °C. IR: *v* 3407, 3292, 3157, 3124 (4NH), 1646 (CO) cm^−1^. ^1^H-NMR: *δ* 2.51(s, 3H, CH_3_), 6.24-7.76 (m, 9H, ArH ), 9.34 (s, 1H, D_2_O exchangeable, NH), 9.83 (s, 1H, D_2_O exchangeable, NH), 10.90 (s, 1H, D_2_O exchangeable, NH), 11.12 (s, 1H, D_2_O exchangeable, NH). MS *m/z* (%): 324 (M^+^, 18), 229 (100), 197 (23), 155 (42), 77 (32). Anal. Calcd for C_17_H_16_N_4_OS (324.10): C, 62.94; H, 4.97; N, 17.27; S, 9.88%. Found C, 62.77; H, 4.94; N, 17.18; S, 9.72%.

### 5.5. Reaction of 1-[(2-methyl-1H-indole)-3-carbonyl]thiosemicarbazide derivatives ***8a,b*** with conc. H_2_SO_4_

A solution of **8a** or **8b** (1 mmol) in conc. sulfuric acid (5 mL) was stirred at room temperature for 4 h. The reaction mixture was poured into cold water. The solid was collected by filtration, washed with water, dried and crystallized from DMF to give product **12a** or **12b**.

*3-(5-Amino-[1,3,4]thiadiazol-2-yl)-2-methyl-1H-indole* (**12a**): Yield 82%; white microcrystals; mp = 162 °C. IR: *v* 3401 (NH), 3281, 3177 (NH_2_) cm^−1^. ^1^H-NMR: *δ* 2.53 (s, 3H, CH_3_), 4.34 (s, 2H, D_2_O exchangeable, NH_2_), 7.06–7.63 (m, 4H, ArH), 11.12 (s, 1H, D_2_O exchangeable, NH); ^13^C-NMR: *δ* 15.13 (CH_3_), 112.55, 119.75, 120.26, 122.36, 123.75, 124.33, 125.75, 129.73, 150.13, 152.57 (Ar-C); MS *m/z* (%): 231 (M^+^ +1, 2), 230 (M^+^, 23), 189 (31), 158 (100), 130 (36), 103 (23), 77 (32). Anal. Calcd for C_11_H_10_N_4_S (230.06): C, 57.37; H, 4.38; N, 24.33; S, 13.92%. Found C, 57.23; H, 4.34; N, 24.18; S, 13.86%.

*3-(5-Phenylamino-[1,3,4]thiadiazol-2-yl)-2-methyl-1H-indole* (**12b**): Yield 82%; white microcrystals; mp = 140 °C. IR: *v* 3401, 3177 (2NH) cm^−1^. ^1^H-NMR: *δ* 2.51 (s, 3H, CH_3_), 6.34–7.74 (m, 9H, ArH), 10.36 (s, 1H, D_2_O exchangeable, NH), 11.12 (s, 1H, D_2_O exchangeable, NH). MS *m/z* (%): 307 (M^+^ +1, 43), 306 (M^+^, 100), 229 (14), 197 (32), 155 (43), 77 (54). Anal. Calcd for C_17_H_14_N_4_S (306.09): C, 66.64; H, 4.61; N, 18.29; S, 10.47%. Found C, 66.54; H, 4.55; N, 18.10; S, 10.39%.

### 5.6. Reaction of 1-[(2-methyl-1H-indole)-3-carbonyl]thiosemicarbazide derivatives ***8a,b*** with potassium hydroxide

A suspension of **8a** or **8b** (1 mmol) in potassium hydroxide solution (5 mL, 7%) in a closed Teflon vessel was irradiated by MW at 400 watt for 5 min. The reaction mixture was cooled then acidified with 10% hydrochloric acid to pH 6. The solid that formed was filtered off, washed with water, dried and finally crystallized from DMF to give product **15a** or **15b**.

*3-(5-Mercapto-4H-[1,2,4]triazol-3-yl)-2-methyl-1H-indole* (**15a**): Yield 76%; white crystals (from DMF); mp = 236 °C. IR: *v* 3401, 3294, 3155 (3NH) cm^−1^. ^1^H-NMR: *δ* 2.52 (s, 3H, CH_3_), 6.11–7.75 (m, 4H, ArH), 9.39 (s, 1H, D_2_O exchangeable, NH), 9.88 (s, 1H, D_2_O exchangeable, NH), 11.09 (s, 1H, D_2_O exchangeable, NH); ^13^C-NMR: *δ* 14.83 (CH_3_), 111.95, 117.75, 120.36, 121.15, 122.16, 123.25, 124.23, 125.25, 155.13, 162.84 (Ar-C); MS *m/z* (%): 231 (M^+^ +1, 6), 230 (M^+^, 100), 158 (98), 130 (19), 155 (32), 77 (12). Anal. Calcd for C_11_H_10_N_4_S (230.06): C, 57.37; H, 4.38; N, 24.33; S, 13.92%. Found C, 57.26; H, 4.32; N, 24.21; S, 13.73%.

*3-(5-Mercapto-4-phenyl-4H-[1,2,4]triazol-3-yl)-2-methyl-1H-indole* (**15b**): Yield 82%; white crystals; mp = 220 °C. IR: *v* 3401, 3177 (2NH) cm^−1^. ^1^H-NMR: *δ* 2.52 (s, 3H, CH_3_), 6.54–7.72 (m, 9H, ArH), 11.10 (s, 1H, D_2_O exchangeable, NH), 13.21 (s, H, D_2_O exchangeable, NH). MS *m/z* (%): 307 (M^+^ +1, 43), 306 (M^+^, 100), 229 (14), 197 (32), 155 (43), 77 (54). Anal. Calcd for C_17_H_14_N_4_S (306.09): C, 66.64; H, 4.61; N, 18.29; S, 10.47%. Found C, 66.52; H, 4.53; N, 18.12; S, 10.53%.

### 5.7. Reaction of 1-[(2-methyl-1H-indole)-3-carbonyl]thiosemicarbazide derivatives ***8a,b*** with mercuric oxide

A solution of **8a** or **8b** (1 mmol) in dimethylformamide (10 mL), containing mercuric oxide (0.216 g, 1 mmol), was irradiated by MW at 400 Watt for 6 min. in a closed Teflon vessel. The reaction mixture was left overnight at room temperature and poured into cold water. The precipitate formed was filtered off, washed with water, dried and finally crystallized from ethanol/DMF to give product **19a** or **19b**.

*3-(5-Amino-[1,3,4]oxadiazol-2-yl)-2-methyl-1H-indole* (**19a**): Yield 84%; white crystals; mp = 188 °C. IR: *v* 3401 (NH), 3284, 3179 (NH_2_) cm^−1^. ^1^H-NMR: *δ* 2.52 (s, 3H, CH_3_), 4.39 (s, 2H, D_2_O exchangeable, NH_2_), 6.11–7.75 (m, 4H, ArH), 11.12 (s, 1H, D_2_O exchangeable, NH). MS *m/z* (%): 215 (M^+^ +1, 44), 214 (M^+^, 100), 229 (19), 197 (32), 155 (42), 77 (68). Anal. Calcd for C_11_H_10_N_4_O (214.09): C, 61.67; H, 4.71; N, 26.15%. Found C, 61.61; H, 4.42; N, 26.00%.

*3-(5-Phenylamino-[1,3,4]oxadiazol-2-yl)-2-methyl-1H-indole* (**19b**): Yield 87%; white crystals; mp = 248 °C. IR: *v* 3404, 3179 (2NH) cm^−1^. ^1^H-NMR: *δ* 2.52 (s, 3H, CH_3_), 6.33–7.78 (m, 9H, ArH), 10.63 (s, 1H, D_2_O exchangeable, NH), 11.12 (s, 1H, D_2_O exchangeable, NH); ^13^C-NMR: *δ* 15.23 (CH_3_), 114.15, 119.75, 121.45, 122.39, 123.85, 124.73, 125.78, 127.96, 128.31, 129.73, 130.61, 132.74, 153.13, 156.57 (Ar-C); MS *m/z* (%): 290 (M^+^, 3.4), 242 (100), 158 (28), 143 (27), 72 (74). Anal. Calcd for C_17_H_14_N_4_O (290.12): C, 70.33; H, 4.86; N, 19.30%. Found C, 70.13; H, 4.80; N, 19.11%.

### 5.8. Synthesis of 3-[(3-methyl-4-phenylhydrazono-5-oxopyrazolin-1-yl)carbonyl]-2-methyl-1H-indole *(**21**)*

*Method A:* To a solution of hydrazide **7** (1.89g, 10 mmol) in DMF (5 mL), ethyl 2-(*N*-phenylhydrazono)-3-oxobutanoate (**20**) (10 mmol), a few drops of glacial acetic acid were added. The reaction mixture was irradiated by MW at 400 Watt for 3 min, and then the reaction mixture was evaporated to half its volume and kept at room temperature overnight. The solid product was collected by filtration, then diluted with water, dried, and recrystallized from ethanol to give **21**. Yield 84%; yellow microcrystals (from ethanol); mp = 328 °C. IR: *v* 1706, 1664 (2C=O), 3407, 3346 (2NH) cm^−1^. ^1^H-NMR: *δ* 2.31 (s, 3H, CH_3_), 2.52 (s, 3H, CH_3_), 6.57-7.89 (m, 9H, ArH), 11.20 (s, 1H, D_2_O exchangeable, NH), 13.11 (s, 1H, D_2_O exchangeable, Hydrazone-NH); ^13^C-NMR: *δ* 14.33 (CH_3_), 35.45 (CH_3_), 113.15, 119.75, 120.26, 121.05, 122.37, 123.79, 124.33, 125.78, 127.16, 128.01, 129.76, 130.61, 152.13, 154.27, 158.57, 159.84 (Ar-C); MS *m/z* (%): 360 (M^+^ +1, 7), 359 (M^+^, 33), 313 (18), 243 (43), 158 (100), 102 (16), 77 (29). Anal. Calcd for C_20_H_17_N_5_O_2_ (359.14): C, 66.84; H, 4.77; N, 19.49%. Found C, 66.74; H, 4.58; N, 19.26%.

*Method B*: *Step 1:Synthesis of 3-[(3-methyl-5-oxopyrazolin-1-yl)carbonyl]-2-methyl-1H-indole* (**22**): A mixture of hydrazide **7** (1.89g, 10 mmol) and ethyl 3-oxobutanoate (10 mmol) in absolute ethanol (20 mL) was irradiated by MW at 400 Watt for 6 min. in a closed Teflon vessel. The reaction mixture was then cooled and the formed precipitate was filtered off, washed with ethanol, and finally recrystallized from ethanol to afford **22**. Yield 77%; yellow microcrystals; mp = 328 °C. IR: *v* 1708, 1664 (2C=O), 3409 (NH) cm^−1^. ^1^H-NMR: *δ* 2.31 (s, 3H, CH_3_), 2.52 (s, 3H, CH_3_), 3.24 (s, 2H, CH_2_), 6.87–7.93 (m, 4H, ArH), 11.21 (s, 1H, D_2_O exchangeable, NH). MS *m/z* (%): 255 (M^+^, 36), 189 (100), 155 (38), 117 (52), 77 (31). Anal. Calcd for C_14_H_13_N_3_O_2_ (255.10): C, 65.87; H, 5.13; N, 16.46%. Found C, 65.80; H, 5.01; N, 16.31%.

*Step 2:Coupling of 3-[(3-methyl-5-oxopyrazolin-1-yl)carbonyl]-2-methyl-1H-indole* (**22**): To a cold solution of compound **22** (2.55 g, 10 mmol) in pyridine (30 mL) was added phenyldiazonium salt solution (10 mmol) [prepared by diazotizing aniline (10 mmol) dissolved in 6M hydrochloric acid (6 mL) with a solution of sodium nitrite (0.7 g) in water (5 mL)]. The addition of diazonium salt was carried out with rapid stirring over a period of 30 min. The reaction mixture was stirred for a further 30 min and left to stand in an ice bath for 12 h, The solid that separated was filtered off, washed with water and finally recrystallized from ethanol to give product proved to be identical in all respects (mp, mixed mp and IR spectra) with the phenylhydrazone **21** which obtained from method A.

### 5.9. Preliminary Antimicrobial Screening

Overnight culture was streaked on the surface of Muller-Hinton agar plate. Sterile filter paper disc was saturated with 10 μL of 0.5 mg/mL w/v solution of the compound under investigation in DMF. The plates and discs were then incubated at 37 °C (for bacteria) and at 28 °C (for fungi) for 24 h and examined for inhibition zones to determine the activity of the tested compounds.

## 6. Conclusions

A number of heterocyclic azoles, incorporating indole moieties, were synthesized using microwave irradiation and evaluated for their biological activities. The results of the antimicrobial activity of the synthesized products were promising.
